# Unlocking the potential of ancient fish DNA in the genomic era

**DOI:** 10.1111/eva.12811

**Published:** 2019-05-28

**Authors:** Tom Oosting, Bastiaan Star, James H. Barrett, Maren Wellenreuther, Peter A. Ritchie, Nicolas J. Rawlence

**Affiliations:** ^1^ School of Biological Sciences Victoria University of Wellington Wellington New Zealand; ^2^ Department of Biosciences, Centre for Ecological and Evolutionary Synthesis University of Oslo Oslo Norway; ^3^ Department of Archaeology University of Cambridge Cambridge UK; ^4^ Department of Archaeology and Cultural History NTNU University Museum Trondheim Norway; ^5^ Trinity Centre for Environmental Humanities Trinity College Dublin Dublin Ireland; ^6^ Nelson Seafood Research Unit Plant and Food Research Nelson New Zealand; ^7^ School of Biological Sciences University of Auckland Auckland New Zealand; ^8^ Otago Palaeogenetics Laboratory, Department of Zoology University of Otago Dunedin New Zealand

**Keywords:** ancient DNA, archaeology, fish, fisheries, genomics, high‐throughput sequencing, next‐generation sequencing

## Abstract

Fish are the most diverse group of vertebrates, fulfil important ecological functions and are of significant economic interest for aquaculture and wild fisheries. Advances in DNA extraction methods, sequencing technologies and bioinformatic applications have advanced genomic research for nonmodel organisms, allowing the field of fish ancient DNA (aDNA) to move into the genomics era. This move is enabling researchers to investigate a multitude of new questions in evolutionary ecology that could not, until now, be addressed. In many cases, these new fields of research have relevance to evolutionary applications, such as the sustainable management of fisheries resources and the conservation of aquatic animals. Here, we focus on the application of fish aDNA to (a) highlight new research questions, (b) outline methodological advances and current challenges, (c) discuss how our understanding of fish ecology and evolution can benefit from aDNA applications and (d) provide a future perspective on how the field will help answer key questions in conservation and management. We conclude that the power of fish aDNA will be unlocked through the application of continually improving genomic resources and methods to well‐chosen taxonomic groups represented by well‐dated archaeological samples that can provide temporally and/or spatially extensive data sets.

## 1 INTRODUCTORY PARAGRAPH

Advances in DNA sequencing technologies and associated downstream analyses over the last decade have transformed research on nonmodel species in evolutionary ecology (da Fonseca et al., 2016). These advancements are now being coupled with improved methods for extracting DNA from archaeological specimens to gain insights into complex historical patterns and processes (Hofreiter et al., 2015). The term ancient DNA (aDNA) refers to DNA derived from plants and animals that have been dead for a prolonged period of time, typically for more than 100 years. The application of improved DNA extraction methods and the subsequent move to ancient genome‐wide sequencing allows exciting, and so far little explored, questions to be investigated with increased power. These questions are manifold and include research topics such as the past distribution of species and their migration patterns (Palkopoulou et al., 2015). If aDNA data can be generated with sufficient resolution and collected across large time scales, then these studies can also reveal past diversification (Nielsen et al., 2017) and introgression events (Huerta‐Sanchez et al., 2014; Vernot & Akey, 2014). Such time series can further be used to investigate changes in genetic variation among populations (Díez‐del‐Molino, Sánchez‐Barreiro, Barnes, Gilbert, & Dalén, 2018; Hofman, Rick, Fleischer, & Maldonado, 2015), and responses to anthropogenic factors and climate change (de Bruyn, Hoelzel, Carvalho, & Hofreiter, 2011; Dalen et al., 2007; Lagerholm et al., 2017). So far, the majority of aDNA research has focused on species in terrestrial ecosystems, such as studies of ancient humans, domestic animals or extinct megafauna (Hofreiter et al., 2015). Significantly fewer studies have focused on fish aDNA, despite the abundance of preserved material. This rich archaeological reservoir holds a fantastic potential for addressing a range of outstanding evolutionary, environmental and taxonomic questions about aquatic species. Here, we synthesize recent developments in the field of aDNA that could be used to explore the evolutionary history of teleost fish species and yield insights into the eco‐evolutionary dynamics of populations over time. We focus on this group because it is the most diverse vertebrate assemblage, occupies the majority of existing habitats on earth, has abundant representatives on most food webs, has been an important resource for humans for millennia and is currently of significant economic interest for aquaculture and wild fisheries. We also believe that the current advances in sequencing technologies will enable the field of fish aDNA to proliferate in the near future, making the focus timely. Lastly, by unlocking the real potential of fish aDNA by moving into the genomic era and capitalizing on the latest DNA extraction, library prep and sequencing technologies, we can begin to gain insights and develop an understanding of evolutionary questions that have relevance to the field of fisheries management, wildlife conservation and practices used for governing biodiversity in general.

## 2 A SHORT OVERVIEW OF FISH ADNA STUDIES

### 2.1 Mitochondrial DNA—the basis for fish aDNA research

**Figure 1:**
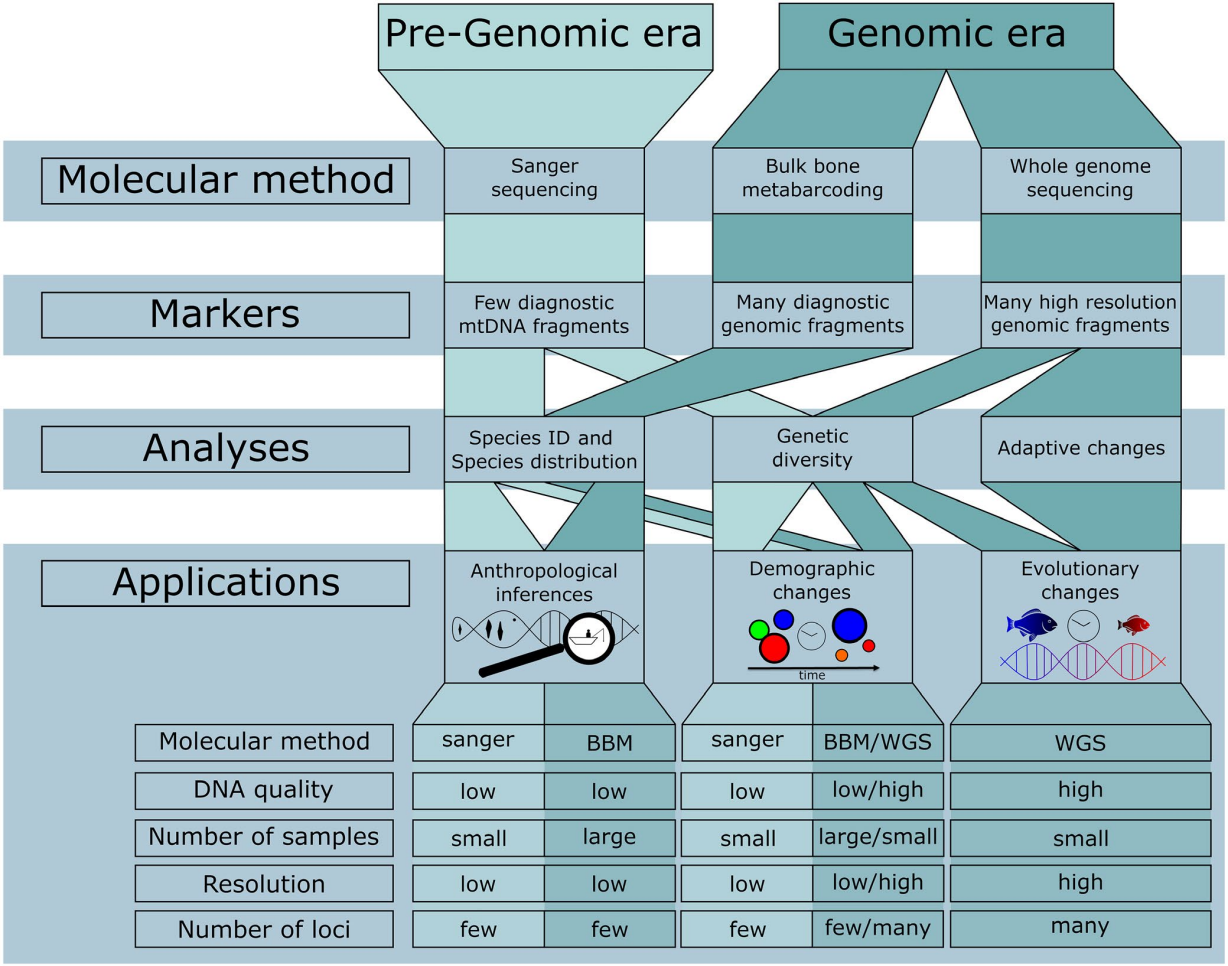
How advances in sequencing technologies will drive applications of fish aDNA. BBM, bulk bone metabarcoding; WGS, whole genome sequencing

A number of studies have targeted mitogenomic (mtDNA) loci using the polymerase chain reaction (PCR) combined with Sanger sequencing to identify species and within‐species diversity, to perform demographic reconstructions and to retrace the geographic origin of traded specimens (Figure 1). Species identification of archaeological bones is a crucial first step for reconstructing past geographic ranges. Nonetheless, morphological identification can be difficult for species with similar phenotypic characteristics or when dealing with degraded samples that have lost their diagnostic characters. In the past, aDNA data have been used to identify specific species of salmon (*Oncorhynchus*spp.) (Yang, Cannon, & Saunders, 2004), sturgeon (*Acipenser*spp.) (Ludwig, Arndt, Debus, Rosello, & Morales, 2009) and other fish species (Kemp & Huynen, 2014; Longenecker et al., 2014; Silva, Malabarba, & Malabarba, 2017; Zivaljevic, Popovic, Snoj, & Maric, 2017). Moreover, the identification of a locally extinct species of sturgeon (*A. sturio*) in the Rhône River provided essential baseline data for the reintroduction of this species in the area (Nikulina & Schmolcke, 2016; Pagès et al., 2008). Similarly, the temporal analyses of mtDNA haplotypes have been used to map the distributional changes of species in response to past climatic change (e.g., salinification, global warming or access to new migration routes) (Ciesielski & Makowiecki, 2005; Splendiani et al., 2016; Wooller, Gaglioti, Fulton, Lopez, & Shapiro, 2015), in one example going as far back as 14,000 years (Splendiani et al., 2016). Such temporal perspectives help provide an understanding of species responses to past climate change, which could be a helpful predictor of adaptations to future climate change. Furthermore, the incorporation of data from aDNA analyses into data sets from contemporary populations enables the demographic features of historic populations to be reconstructed more precisely. In some cases, temporal sampling can be used to replace key assumptions that underlie a model with empirical information. Reconstruction of genetic diversity of >3,000‐year‐old Chinook salmon (*O. tshawytscha*) in the Columbia River basin identified the loss of genetic variation prior to human arrival (Johnson, Kemp, & Thorgaard, 2018). Using a similar approach, 1,500‐year‐old Atlantic cod (*Gadus morhua*) samples from Iceland were used to reconstruct population abundance trends, identifying higher levels of genetic diversity in the historic population (Olafsdottir, Westfall, Edvardsson, & Palsson, 2014). Metcalf et al. (2012) used up to 155‐year‐old cutthroat trout (*O. clarkia*) samples collected prior to historical stocking in North America, compared them with contemporary samples and showed a history of lineage extinction, taxonomic errors and range changes as a result of human movement and stocking activities. Finally, ancient mtDNA has provided novel findings about the cultural use of fish and traced early trading routes, highlighting the value of aDNA in anthropological and archaeological research (Arndt et al., 2003; Grier et al., 2013; Speller, Yang, & Hayden, 2005). These studies have demonstrated how temporally spaced sampling using aDNA methods is able to provide a range of new insights, which cannot be obtained using contemporary samples alone. However, due to technological and economic constraints associated with retrieving aDNA data, these studies have been limited to using mitochondrial markers and sometimes investigating a relatively small number of individuals.

### 2.2 Moving towards genome‐wide inference

Over the last few years, fish aDNA studies have started to report results from genome‐wide sequence data. A notably early study reported loss of genetic variation in Atlantic Salmon (*Salmo salar*) using microsatellite data obtained from 60‐year‐old scales (Nielsen, Hansen, & Loeschcke, 1997). More recently, aDNA data based on a diverse set of genetic markers (i.e., mtDNA, microsatellites and nuclear single nucleotide polymorphisms, SNPs) provided evidence of distinct past Pacific herring (*Clupea pallasi*) populations, implying that the contemporary populations represent only a fraction of a previous metapopulation that was more abundant and genetically diverse (Speller et al., 2012). Similarly, Hutchinson, Oosterhout, Rogers, and Carvalho (2003) used Atlantic cod (*G. morhua*) otoliths from the North Sea and showed a significant reduction in genetic diversity, strong genetic drift and population replacement between 1954 and 1998 in one fisheries stock. By genetically determining the sex of ancient fish remains, the effects of past sex‐selective fishing strategies can also be investigated (Royle et al., 2018). By targeting a set of 28 informative nuclear SNPs, the geographic origin of Atlantic cod (*G. morhua*) samples recovered from the Mary Rose, a late mediaeval (AD 1545) navy shipwreck in the United Kingdom (Hutchinson et al., 2015) was determined. Combined with stable isotope analyses, the aDNA data showed that the cod had come from different areas, possibly ranging from Newfoundland to Atlantic Europe and Arctic Norway. This finding corroborated historical evidence that the globalization of commercial fishing started as early as the 16th Century. By using whole genome sequencing approach, Atlantic cod bones excavated from Haithabu in Germany were shown to originate from the far north of Norway (Star et al., 2017). This study analysed a SNP data set of 156,695 loci, allowing for the population assignment of fish by identifying specific combinations of chromosomal inversion genotypes among cod populations. Using historic samples, Therkildsen, Hemmer‐Hansen, Als, et al. (2013a) screened >1,000 gene‐associated genomic SNPs in Atlantic Cod (*G. morhua*) and identified 77 SNPs that showed high levels of differentiation over a temporal scale. Interestingly, changes in allele frequency at different loci were correlated with either temperature or probabilistic maturation reaction norm (PMRN), a commonly used measurement for identifying changes in maturation rate (Heino, Diaz Pauli, & Dieckmann, 2015; Heino & Dieckmann, 2008). The authors were then able to relate changes in life‐history traits to specific loci, possibly associated with adaptive changes in response to overexploitation (Heino, Dieckmann, & Godo, 2002). Finally, studies have used a bulk bone metabarcoding (BBM, further discussed below) approach for the large‐scale identification of fish species from morphologically indistinguishable bone fragments. This has helped to provide a more complete view of the zoo‐archaeological records from past coastal communities (Douglass et al., 2018; Grealy et al., 2016), thus allowing for more accurate reconstruction of species past geographical distribution (Seersholm et al., 2018).

The relatively young field of fish aDNA has recently been advanced by a number of insights, namely (a) that DNA can be preserved for long time periods and (b) high‐quality genomic data can be obtained from ancient fish remains, and now questions can be addressed about how populations of aquatic species have changed over time. Recent studies targeting multiple genomic loci through high‐throughput sequencing (HTS) are indicative of the direction the field of fish aDNA is heading, although several challenges remain for studies of fish aDNA. Below, we discuss how advances in molecular methods will progress the field of fish aDNA, describe key questions in fish ecology, evolution and resource management that are able to be addressed, emphasize the need for interdisciplinary collaborations and present a future perspective including challenges and priorities. By doing this, we aim to show the potential of this emerging field.

## 3 FISH ANCIENT DNA IS MOVING INTO THE GENOMIC ERA

One of the main limitations for advancing fish aDNA research is sample preservation. Fish bones are brittle, porous and very light, which is generally associated with poor DNA preservation because biomolecules are not as well isolated from its environment compared with other types of bone. DNA degradation is influenced by a wide range of variables, making it extremely difficult to predict which archaeological sites contain high‐quality samples for genomic analyses. The aquatic environment in particular exposes biological material to a range of chemicals that cause acidification and oxidation of compounds. Generally, cold and dry environments preserve DNA better compared with hot and humid environments (Boessenkool et al., 2017; Damgaard et al., 2015; Hansen et al., 2017; Pinhasi et al., 2015; Willerslev, Hansen, & Poinar, 2004). Also, dense soil (e.g., clay) has been shown to preserve biological material well (Hlinka, Ulm, Loy, & Hall, 2002), though this may not be present in coastal archaeological sites characterized by harsh, acidic, preservation conditions. Conversely, exposure to high heat (e.g., cooking or boiling) alters the physical condition of biological material, damaging the DNA, potentially rendering the sample unsuitable for aDNA analyses. Several recent methodological advances have revolutionized our ability to utilize fish aDNA and investigate the genetic population structure and demographic history of natural populations.

First, the development of improved aDNA extraction methods has greatly increased the number of samples suitable for genome‐wide sequencing (Boessenkool et al., 2017; Dabney et al., 2013; Damgaard et al., 2015; Gondek, Boessenkool, & Star, 2018; Hansen et al., 2017). Such enhanced methods allowed for the extraction of high‐quality aDNA from archaeological fish bone from a wide range of species, preservation conditions and ages. For instance, DNA (including nuclear DNA) has been consistently amplified from archaeological fish bone up to 10,000 years old (Speller et al. (2012), sometimes in stretches for up to 250 bp (Seersholm et al., 2018), and even includes amplification from bones retrieved from warm tropical locations (Douglass et al., 2018; Grealy et al., 2016). This consistent success of DNA retrieval shows that archaeological and palaeontological fish bone is a previously unrecognised source of potentially high‐quality aDNA. Other proven DNA sources from fish consist of scales or otoliths that have been stored in collections to inform fisheries management (e.g., Hauser, Adcock, Smith, Ramirez, & Carvalho, 2002; Nielsen et al., 1997; Therkildsen, Hemmer‐Hansen, Als, et al., 2013a), although here the temporal perspective is often limited to the last century.

Second, while most ancient bones have <5% endogenous DNA (e.g., Noonan et al., 2005) (with a few exceptions, e.g., Meyer et al., 2012), fish bones have been found to carry a high amount of endogenous DNA (Star et al., 2017), something that needs to be considered when conducting whole genome sequencing. Boessenkool et al. (2017) and Star et al. (2017) showed ancient Atlantic cod (*G. morhua*) bones over 1,000 years old can contain between 15% and 50% endogenous DNA content. This observation is remarkable, given that high levels of endogenous DNA are usually associated with higher density bones (Geigl & Grange, 2018), such as the petrous bone in mammals (Gamba et al., 2014). Combined, high levels of endogenous DNA, advances in HTS (further discussed below), and the relative small size of fish genomes (often <1 Gbp), implies that generating genomic aDNA for fish is becoming accessible to a wide scientific community. Endogenous DNA content can be increased using capture enrichment, where custom DNA baits (generally designed from a reference genome from the study species or a close relative) hybridize with complimentary sequences and subsequently separated from foreign DNA (Carpenter et al., 2013; Hofreiter & Shapiro, 2012). However, such applications can be time and cost expensive.

Finally, advances in HTS has made the genome‐wide acquisition of thousands to millions of genetic markers—including rare variants—increasingly affordable for nonmodel organisms (Figure 1) (Mardis, 2008). Such genome‐wide DNA sequencing provides an unparalleled opportunity to detect fine‐scale differentiation in otherwise homogenous populations and species (Barth et al., 2017; Martin et al., 2018; Milano et al., 2014). Another novel approach is bulk bone metabarcoding (BBM) which simultaneously analyses hundreds of nondiagnostic specimens for species identification (Grealy et al., 2015; Haouchar et al., 2014; Murray et al., 2013). BBM is thus a useful technique to quickly assess the level of DNA preservation in archaeological sites across a range of species. BBM is especially useful in fish as fragmentary cranial and postcranial bones can be difficult to identify in teleost species. Indeed, while morphological identifications of complete bones may be possible to family level, genus and species level assignments may not be possible due to the absence of distinct morphological characters. Incorporating aDNA and BBM methods would exponentially increase the research questions that archaeological fish bone can answer. For instance, a combination of both methods would allow to reconstruct prehistoric fishing zones in tropical reef contexts with high regional biodiversity (Giovas, Lambrides, Fitzpatrick, & Kataoka, 2017), or temporal changes in the range of marine habitats that are exploited through fishing. Seersholm et al. (2018) used BBM and found genetic evidence for the presence of both freshwater (*Anguilla australis*) and marine (*Conger* sp. and *Gnathophis* sp.) eels at archaeological sites from New Zealand's indigenous Māori. Although eels were known to be an important traditional food source for Māori, this is the first time such remains have been identified due to their poor preservation in the archaeological record. This study shows how BBM can be a valuable tool that complements more traditional archaeological and palaeontological approaches as it is able to identify species without the need for traditional morphological characteristics. Methodological advances in aDNA, including the use of “rescue PCR” (Johnson et al., 2018) and PCR enhancers (e.g., PEC‐P; Zhang, Kermekchiev, & Barnes, 2010) for suboptimal samples, should be combined with BBM to increase the power of this technique. Using these techniques, Palmer, Tushingham, and Kemp (2018) were able to better understand subsistence fishing practices, especially those of small forage fish, of indigenous prehistoric communities in northern California.

## 4 GENOMIC ADNA ANALYSES TO INFORM FISHERIES MANAGEMENT AND CONSERVATION

The novel applications of fish aDNA now allow us to better understand how fish stocks have changed over time, both in terms of natural variation in abundance and range, and from the anthropogenic impacts of fishing. The rise of industrial‐scale fishing during the 1950s and the lack of any significant genetic sampling of stocks prior to the 1970s mean it has been difficult to get an accurate picture of how harvesting has affected the genetic diversity and distribution of fisheries species. A key goal of research for fisheries management is to detect and ultimately reduce the negative effects of overexploitation (Ovenden, Berry, Welch, Buckworth, & Dichmont, 2015; Ward, 2000), so that species can be fished sustainably over long time periods. The advantage of aDNA is that it represents a sample of the fish stock before the onset of commercial fishing, which can be compared with samples taken from a contemporary fish stock. Temporal population sampling could enable testing for the loss of genetic diversity due to stock depletion and the genetic effects of size‐selective harvesting (Allendorf, England, Luikart, Ritchie, & Ryman, 2008), and whether the stock structure and distribution has changed over time (Perry, Low, Ellis, & Reynolds, 2005). Understanding changes to genetic diversity from sampling past populations and comparing them to contemporary populations can thus provide invaluable information about how genetic baselines may be shifting. Fisheries managers could then adjust fishing pressures in certain areas to prevent further loss of genetic diversity. Furthermore, an understanding of population responses to earlier climate change events could provide important insights about the possible changes that will happen to stocks in the future.

Evidence for the loss of genetic diversity from the depletion of a fish stock has been reported for Australasian snapper (*Chrysophrys auratus*) (Hauser et al., 2002), Atlantic salmon (*S. salar*) (Nielsen et al., 1997) and North Sea cod (*G. morhua*) (Hutchinson et al., 2003), using aDNA extracted from dried scales and otoliths. The temporal nature of aDNA samples can be used to test for a loss of genetic variation as a consequence of stock depletion over time. One prediction from prolonged fishing pressure is that the strength of genetic drift is predicted to increase and eliminate alleles in a population if there has been a significant population size reduction due to overexploitation (Ovenden et al., 2016). This is supported by the finding that the allelic richness appears to be lower in exploited species compared with species that are not heavily affected by fishing (Pinsky & Palumbi, 2014). A prefishing sample point provided by aDNA would be a direct test of whether an exploited population had lost allelic diversity. The recent increase of genome‐wide sequencing data (Star et al. (2017) from ancient samples presents a new opportunity for conducting genome scans for adaptive loci (Bernatchez & Wellenreuther, 2018). Ancient and contemporary sampling could be used to discover loci that have been subjected to selection as a result of the intense size‐selective force that industrial‐level fishing as applied to stocks over the last 50 years. This type of selection is expected to produce smaller fish that reach maturation earlier (Heino et al., 2015). As more genomic information becomes available from studies of quantitative trait loci (QTL) and genome wide association studies (GWAS) (Ashton, Ritchie, & Wellenreuther, 2017), the important loci underlying fish growth rates and maturation could also be more directly investigated.

It has been well documented that past climate change had an impact on the structure and distribution of marine populations (Perry et al., 2005). In particular, the preferred sea temperature for a species is a strong factor that determines range and dispersal success (Poloczanska et al., 2013). The range and distribution of many fish species will shift in response to future ocean warming, and fisheries management will need to adapt to meet the challenge of protecting new emerging stocks and adjust to lower sustainable yields from traditionally productive stocks. However, the genetic patterns and evolutionary process involved in past climate‐driven range shifts in the marine realm are largely unknown, which means there is a gap in information that could be used to support the development of a framework for adapting future fisheries management to climate change. New methodologies (e.g., BBM) utilizing large sample sizes will enable a species' past range to be estimated and compared with the contemporary range. Moreover, as climate‐driven range shifts are detected in modern populations it might be possible to make comparisons with past populations (Ramos et al., 2018), as long as there have been no cryptic biological turnover events in the archaeological and palaeontological records (Collins et al., 2014). However, for these types of inferences it could be difficult to distinguish among the different selective scenarios, for example, natural versus anthropogenic processes, particularly given that many of these processes often work in concert. Genome‐wide sequencing of aDNA samples could be used to test for adaptive loci associated with range expansions and how often fish with k‐selected life‐history traits moved compare to those species with late maturation and larger body size (McLean et al., 2018). Overall, our understanding of the impacts of fishing and climate has much to gain from temporal sampling of exploited species using aDNA approaches.

## 5 INTERDISCIPLINARY RESEARCH AT THE INTERFACE OF ARCHAEOLOGY AND BIOLOGY

One outcome of the advances in genomic approaches and aDNA research is the convergence of biology, archaeology and conservation management. The integration of these fields can concurrently help answer questions of interest to both fields. As presented above, biology has assisted archaeological research by being able to identify samples to species, or even population level from nondiagnostic remains, providing novel insight regarding location and/or seasons of resource extraction (Bulter, 1998; Ewonus, Cannon, & Yang, 2011; Grier et al., 2013; Star et al., 2017). Likewise, the intersection of aDNA and archaeology, in the rapidly growing field of conservation archaeo‐genomics (Hofman et al., 2015), has provided conservation authorities with vital baseline data for the management of fisheries stocks (Moss, Rodrigues, Speller, & Yang, 2016; Rodrigues, McKechnie, & Yang, 2018). While species identification is still an important application of molecular biology in archaeology, methods such as ZooMS for protein barcoding are often applied (Harvey, Daugnora, & Buckley, 2018; Hendy et al., 2018). ZooMS entails the comparison of peptide mass fingerprints derived by mass spectrometry, allowing identifications to genus or species level that, like identifications using aDNA, can inform our understanding of past fishing and its ecological impacts. Genomic research on fish bones can be powerfully combined with traditional zoo‐archaeology and stable isotope analyses to infer the relationship between past human populations and changes in biogeography, the intensity of human exploitation, and the development of commodification and long‐range trade (Barrett, 2019). Moreover, the application of environmental aDNA is opening the potential to see taxa that are otherwise invisible, in lake sediments and archaeological deposits (Hebsgaard et al., 2009; Pedersen et al., 2015; Rawlence et al., 2014).

Key insights from archaeological work also greatly enhance the potential of biological research from aDNA. In archaeology, context is everything. Knowing the chronology and depositional context of a subfossil is fundamental to its interpretation. Accurate dating of biological samples can help estimate mutation rates and in turn allow for accurate demographic history assessments (Lambert et al., 2002). Moreover, an awareness of which specimens might represent human translocations, such as the spread of carp aquaculture (Hoffmann, 1994), or the long‐range trade of foods like dried cod (*G. morhua*) or salted herring (*C. pallasi*) (Barrett, 2016). Such findings are as critical to evolutionary inferences regarding biogeography as it is to archaeological and historical interpretation. Changes in the aquatic environment, such as temperature, with potential evolutionary implications, can also be demonstrated by research within archaeology. This palaeo‐ecological information can be derived from study of fish remains themselves (Geffen et al., 2011) and from associated materials such as marine shells (Surge & Barrett, 2012).

## 6 A FUTURE PERSPECTIVE: CHALLENGES AND PRIORITIES

We have proposed that the recent insights in aDNA methodology and sequencing technologies will enable the field of fish aDNA to move into the genomic era and will experience significant growth.

A key challenge will be to integrate genomic and archaeological efforts in the future within a collaborative framework. Overall, the integration of genomic and archaeological approaches has the potential to illuminate a range of questions that cannot be fully addressed by either subject in isolation. When did fisheries‐induced evolution first emerge within a given taxon? When and where were widely distributed species first harvested or traded over long distances? When and where were fish first translocated for the purposes of stocking? To what degree were past changes in fish biogeography caused by human impact versus natural environmental change? This list of questions will grow as interdisciplinary research proceeds. The vast archaeological record holds much more than what can be discovered from aDNA alone, and close relations between archaeology and biology will surely benefit both sciences.

Collaboration with archaeologists will also allow more informed sampling choices to be made, given specimens from most archaeological sites are affected by anthropogenic (e.g., trade vs. processing sites) and taphonomic biases. Such human‐induced bias (e.g., species overrepresentation, selection of certain phenotypes such as size or behavioural traits) should be taken into account when interpreting results. It should be noted that sample size limitations can become a significant issue, as high‐quality samples required for whole genome sequencing may be sparsely distributed for some taxa. Especially, studies which advocate conservation management decisions will require significant spatial or temporal sampling in order to enforce legislation. Applications such as BBM and hybridization capture enrichment do provide some flexibility in the sample quality.

We further wish to highlight that future efforts should focus on expanding the genomic resources that are available for fish, as the vast majority of them have limited or no genomic resources. This is going to be crucial, as teleost fish are represented by over 33,000 extant species, making them the most diverse vertebrate group on the planet (Eschmeyer, Fricke, & Laan, 2014) and, at the time of our review, genome assemblies are only available for 59 fish species (NCBI). Having a well‐assembled reference genome for a study species, or at least of a phylogenetic closely related species, is an important first step that greatly improves the power of aDNA studies and reduces the cost for analysing thousands of loci (Star et al., 2017). Moreover, the limited number of teleost genomes hampers the development of species‐specific barcodes that are of sufficient short length so that they can be used in high‐throughput environmental and palaeo‐environmental DNA approaches (Hanfling et al., 2016; Rawlence et al., 2014; Seersholm et al., 2018). With those considerations, we believe that this is an exciting time for the field of fish aDNA and are convinced that it will contribute to crucial insights to support evidence‐based decisions in fisheries management, conservation and invasion biology.

## CONFLICT OF INTEREST

None declared.

## Data Availability

No original data has been generated for this manuscript.
